# Liver Cirrhosis and Sarcopenia from the Viewpoint of Dysbiosis

**DOI:** 10.3390/ijms21155254

**Published:** 2020-07-24

**Authors:** Hiroki Nishikawa, Hirayuki Enomoto, Shuhei Nishiguchi, Hiroko Iijima

**Affiliations:** 1 Department of Internal Medicine, Division of Gastroenterology and Hepatology, Hyogo College of Medicine, Nishinomiya 6638136, Japan; enomoto@hyo-med.ac.jp (H.E.); hiroko-i@hyo-med.ac.jp (H.I.); 2 Center for Clinical Research and Education, Hyogo College of Medicine, Nishinomiya 6638136, Japan; 3 Kano General Hospital, Osaka 1355118, Japan; nishiguchi@heartfull.or.jp

**Keywords:** liver cirrhosis, skeletal muscle, sarcopenia, gut–liver axis, dysbiosis

## Abstract

Sarcopenia in patients with liver cirrhosis (LC) has been attracting much attention these days because of the close linkage to adverse outcomes. LC can be related to secondary sarcopenia due to protein metabolic disorders and energy metabolic disorders. LC is associated with profound alterations in gut microbiota and injuries at the different levels of defensive mechanisms of the intestinal barrier. Dysbiosis refers to a state in which the diversity of gut microbiota is decreased by decreasing the bacterial species and the number of bacteria that compose the gut microbiota. The severe disturbance of intestinal barrier in LC can result in dysbiosis, several bacterial infections, LC-related complications, and sarcopenia. Here in this review, we will summarize the current knowledge of the relationship between sarcopenia and dysbiosis in patients with LC.

## 1. Introduction

### 1.1. Gut–Liver Axis and Dysbiosis

**Figure 1:**
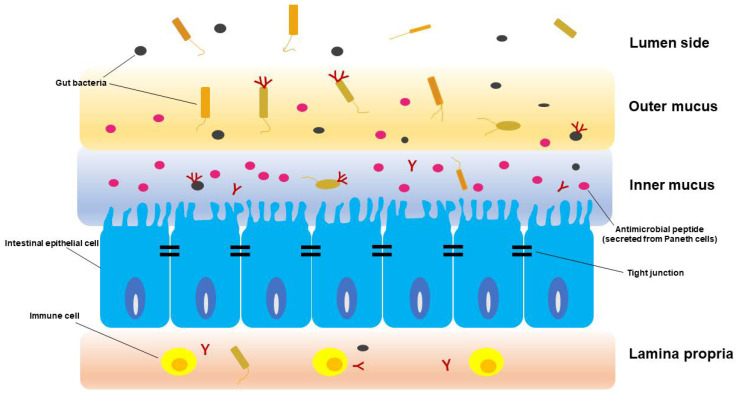
Defensive mechanism of intestinal barrier.

In the gastrointestinal mucosa, various immune cells including macrophages, dendritic cells, etc., are constantly present [1,2]. Paneth cells, which are a type of intestinal epithelial cell, secrete antimicrobial peptides and are responsible for intestinal innate immunity by eliminating pathogens and by symbiosis with resident bacteria [3]. However, the barrier mechanism formed in the mucus layer may be incomplete, and indigestible proteins, bacteria, viruses, etc., along with nutrient components, can always enter the tissue across the mucus barrier [1,2]. In that case, a secondary barrier consisting of macrophages, T cells, and B cells present in the lamina propria will respond to their invasion (the biological barrier, Table 1 and Figure 1). The foreign substances passing through the lamina propria can enter the bloodstream and reach the liver via the portal vein. A large number of Kupffer cells (liver macrophages) are present in the sinusoidal blood vessels of the liver (the final barrier) to create a unique immune system [4]. In this way, the gastrointestinal tract and liver cooperate to participate in biological defense (gut–liver axis) [4,5]. On the other hand, it has been revealed that various intestinal bacteria inhabit the colon, and they play an important role in maintaining homeostasis of the human body (the environmental barrier, Table 1 and Figure 1) [6]. The majority of bacteria in the colon are tightly attached to the outer side of the mucus layer, and the inner side of the mucus layer forms a barrier which limits bacterial contact with the epithelium (the physical barrier, Table 1 and Figure 1) [7]. Dysbiosis refers to a state in which the diversity of gut microbiota (GM) is decreased by decreasing the bacterial species and the number of bacteria that compose the GM [8,9,10,11]. Analysis of GM at the gene level using a next-generation sequencer has come to the fore, and thus GM in patients with various diseases has been analyzed [12]. Not only in *Clostridium difficile* infection and inflammatory bowel diseases [13,14], but also in disorders other than in the gastrointestinal tract including obesity [15], allergy [16], asthma [17], autism [18], and autoimmune diseases [19], it has been pointed out that the GM is disturbed and the diversity is decreased (i.e., dysbiosis).

### 1.2. Sarcopenia and Liver Cirrhosis

In individuals with chronic liver diseases (CLDs), metabolic or nutritional dysfunctions including protein–energy malnutrition (PEM) or muscle abnormalities are frequently found, which can be related to disabilities, poor quality of life, or mortality [20,21,22,23,24,25,26,27,28,29,30]. Liver cirrhosis (LC) involves a hypermetabolic state with increasing demand for calories and protein [23]. In addition, the energy metabolism of LC patients is in a hypercatabolic state, and when fasting early in the morning, they are in the same degree of starvation as when a healthy person fasts for 2–3 days [31,32]. When liver function worsens, the detoxification of harmful substances such as ammonia can be reduced [32]. Branched-chain amino acids (BCAAs) are often excessively consumed in skeletal muscles to detoxify harmful substances in patients with decreased liver function [20,32]. In LC patients, it is difficult to adequately supplement BCAAs with diet intake alone [32]. In LC patients, sarcopenia, which is defined by decline in muscle mass and strength and/or physical activity, can occur because the excessive consumption of BCAAs makes it difficult to synthesize the protein required for muscle mass increase [20]. Sarcopenia is one of the most common consequences seen in patients with LC [20,27,33,34,35,36,37,38,39]. In Japan’s aging population, CLD is also a crucial public health issue because aging is also closely linked to sarcopenia [40,41,42]. How sarcopenia is related to adverse consequences requires looking at sarcopenia as a systemic disorder [22,43,44]. LC-related complications themselves such as hepatocellular carcinoma (HCC), ascites, spontaneous bacterial peritonitis (SBP), varices, hepatic encephalopathy (HE), and acute or chronic liver failure (ACLF) can cause sarcopenia [22,40]. Clinical and research interest in sarcopenia in CLDs has thus been growing internationally. In 2016, the Japanese society of hepatology (JSH) created their own criteria for the assessment of sarcopenia in CLDs [40]. In the JSH criteria for sarcopenia, age limitation is excluded because CLDs can cause secondary sarcopenia due to PEM, which can occur regardless of age. In addition, the measurement of walking speed was abolished due to the difficulty of measuring it in daily clinical practice, and it was decided to use only grip strength for the evaluation of muscle strength (cutoff values: <26 kg in males and <18 kg in females). Furthermore, since computed tomography (CT) is frequently used in CLD patients, a standard value for CT was set for measuring muscle mass, and it was decided to use the bioimpedance analysis method (cutoff values: <7.0 kg/m^2^ in males and <5.7 kg/m^2^ in females) and/or CT method at the L3 level (cutoff values: <42 cm^2^/m^2^ in males and <38 cm^2^/m^2^ in females) to evaluate muscle mass [40]. In Japan, a lot of debate regarding sarcopenia in CLDs has taken place based on the JSH criteria.

Here in this review, we will summarize the current knowledge of the relationship between dysbiosis and sarcopenia in patients with LC.

## 2. Liver Cirrhosis, Hepatic Encephalopathy, and Sarcopenia: Mechanisms and Clinical Impact

LC patients with hyperammonemia are often encountered in routine clinical practice. Most of the ammonia produced in vivo is derived from the digestive tract. The organs that metabolize ammonia include the liver, skeletal muscles, brain, and kidneys. Of these, the only organ with sufficient capacity to detoxify ammonia produced in the body into urea is the liver, which has a urea cycle [45]. LC patients manifest the characteristics of low levels of BCAAs due to PEM and elevated blood ammonia level due to an impaired urea cycle caused by zinc deficiency, etc. [46]. Ammonia suppresses phosphorylation of eukaryotic initiation factor 2α and mammalian target of rapamycin complex1 (mTORC1) signal through general control nonderepressible 2 which is an amino acid deficiency sensor, and directly suppresses protein synthesis in skeletal muscles. BCAA suppresses these reactions and promotes muscle protein synthesis, but L-leucine (one of BCAAs) is consumed due to ammonia metabolism in skeletal muscles [47]. When the L-leucine level is decreased, protein synthesis in skeletal muscles becomes unsuccessful [48].

In LC patients, muscle proteolysis is stimulated via the activation of the ubiquitin-proteasome pathway [49,50]. Persistent chronic inflammation in LC can cause the marked elevation of the pro-inflammatory cytokines including tumor necrosis factor-alpha (TNF-α) and IL-1, -6, which in turn stimulate muscle autophagy [51]. The inflammation-inducible ubiquitin-proteasome system can be linked to muscle atrophy through activation of muscle atrophy-related genes [52]. Myostatin suppresses muscle satellite cell proliferation and differentiation. Elevated myostatin levels in skeletal muscles can cause sarcopenia in LC patients [53]. Hyperammonemia has been demonstrated to elevate muscle myostatin expression via TLR-independent nuclear factor kappa beta activation in an animal model [54]. As serum and skeletal muscle ammonia levels are often elevated in LC because of portosystemic shunts or impaired ureagenesis, significant increase of myostatin expression in skeletal muscles can be observed [55]. The decrease in serum free testosterone levels, BCAA, and insulin-like growth factor-1 levels also result in elevated myostatin levels in LC [56,57]. LC patients are often involved in gonadal dysfunction, which can also result in hypermyostatinemia in skeletal muscles [57]. In our previous report, we demonstrated that elevated serum myostatin level can be associated with hyperammonemia (correlation coefficients; *r* = 0.5856 in males and *r* = 0.3922 in females), hypoalbuminemia (correlation coefficients; *r* = −0.3844 in males and *r* = −0.3945 in females), and poor outcomes [58]. In addition, we found a close inverse correlation between serum myostatin level and psoas muscle mass as assessed by CT at the L3 level in LC patients (median psoas muscle index (high vs. low serum myostatin group); 4.84 cm^2^/m^2^ vs. 6.37 cm^2^/m^2^ (*p* < 0.0001) in males and 3.87 cm^2^/m^2^ vs. 4.25 cm^2^/m^2^ (*p* = 0.0175) in females) [58].

Sarcopenia in LC patients can contribute to increase risk of minimal or overt HE. Hanai et al. reported that in their 120 LC patients sarcopenia (hazard ratio (HR) = 3.31, 95% confidence interval (CI) = 1.19-9.42; *p* = 0.02) and serum BCAA levels <327 nmol/mL (HR = 2.98, 95% CI = 1.08–8.34) were found to be independent adverse predictive factors for the incidence of minimal HE [59]. A recent meta-analysis (6 studies, comprising 1795 patients) reported that sarcopenia was closely linked to the presence of HE (HR = 2.74, 95% CI = 1.87–4.01) [60]. On the other hand, chronic use of proton pump inhibitors (PPIs) can alter the GM and can be a risk factor for HE in LC patients [61,62].

## 3. Dysbiosis and Sarcopenia from the Viewpoint of Nutrition and Metabolism

Skeletal muscle is considered to be a metabolic organ, which consumes a lot of energy and plays an important role in supporting exercise capacity and regulating body fat mass and blood glucose levels. Skeletal muscles take up glucose and control blood glucose levels and play an important role in storing glucose as glycogen [63,64]. Skeletal muscle has also been found to have a role as an endocrine organ secreting myokine (physiologically active substance) and regulating organ function throughout the body [65,66]. Skeletal muscle decline can be associated with insulin resistance and disturbance of GM (muscle–gut axis) [66,67].

Microorganisms in the gut exert their functions mainly through enzyme pathways for the purpose of digesting complex dietary carbohydrates and proteins [68,69]. GM provides the BCAAs including valine, leucine, and isoleucine, and particularly glycine, which is necessary for the synthesis of glutathione. Glutathione has an auxiliary role in protecting cells from reactive oxygen species such as free radicals and peroxides [68,69]. Intake of a high fat diet can cause dysbiosis, which may be linked to the development of colorectal cancer (CRC) [70].

Microbial metabolites from the intestines have been demonstrated to act as nutrients or metabolic modulators in skeletal muscles [71]. Short-chain fatty acids (SCFAs) include organic acids such as butyrate (4 carbon atoms), propionate (3 carbon atoms), and acetate (2 carbon atoms), which are produced by the GM. Acetate and propionate pass into the bloodstream and are taken up by the peripheral organs and the liver, where they can act as substrates for gluconeogenesis and lipogenesis [69,72]. Butyrate has a role in providing energy for cell metabolism and regulates apoptosis, cell differentiation, and chemical modification of nuclear proteins and nucleic acid through action on numerous cells [72,73]. Butyrate is the most important energy source for intestinal epithelial cells and exerts excellent physiological effects such as an anti-inflammatory action. Butyrate has been reported to have a significant effect on skeletal muscles [74,75]. Butyrate can help prevent skeletal muscle mass loss and maintain skeletal muscle mass via anti-inflammatory effects and activation of regulatory pathways, leading to ATP increase and suppression of muscle protein catabolism and apoptosis [73,76]. The relationship between GM and lipid metabolism in CLDs depends on the degree of liver damage [77]. A negative correlation between hepatic venous pressure gradient (HVPG) and butyrate levels in LC patients can be found [78]. Acetate has also been reported to have the following effects: (1) decrease in intestinal pH, (2) suppression of ammonia-producing bacterial growth, and (3) suppression of absorption of intestinal ammonia [79,80]. These observations may account for the high prevalence of sarcopenia in LC patients. The frequency of sarcopenia in LC patients is reported to be 10–70% [40].

## 4. Dysbiosis, Intestinal Permeability, Tight Junction, and Sarcopenia

The function of GM and its associated immune regulation mechanism have been elucidated, and it has been revealed that the intestine, which acts as a barrier at the forefront of the living body, affects the whole body by controlling substance permeation from the gastrointestinal tract into the systemic circulation [71].

It has been revealed that the tight junction (TJ) between intestinal epithelial cells regulates the immune response to invasion of bacteria and foreign antigens from the intestinal tract in cooperation with intestinal-associated lymphoid tissue and the intestinal neuroendocrine network (the physical barrier, Table 1 and Figure 1) [81,82,83]. The major role of epithelial cells in contact with the outside is to protect the host from foreign antigens by forming epithelial cell sheets (tightly adhering cells) with TJs [84]. Furthermore, it has also been revealed that the GM is deeply involved in the development and maintenance of the intestinal epithelial barrier [81,82,83]. TJ was identified as a 47kDa protein and named Zonulin. Zonulin enhances intestinal permeability, is involved in innate immunity, and is strongly suggested to be associated with the development of autoimmune diseases such as type 1 diabetes [81,82,83].

Cumulative evidence has highlighted the relevance of increase in intestinal permeability (i.e., leaky gut syndrome) and consequent bacterial translocation in the development of CLDs. Particularly, in recent hypotheses regarding patients with non-alcoholic fatty liver disease (NAFLD), intestinal permeability impairment, dietary habits, and gut dysbiosis are considered to be the main pathogenic triggers [85,86,87]. Leaky gut is associated with chronic inflammation [87]. In advanced liver diseases, intestinal permeability can be enhanced by portal hypertension, which consequently leads to increased bacterial translocation that further damages liver function. Furthermore, these pathogenic mechanisms are implicated in most LC-related complications, such as SBP, hepatorenal syndrome, severe ascites, HE, sarcopenia, and HCC [85,86,87]. In LC rats, intestinal bacteria such as Gram-negative bacilli in mesenteric lymph nodes were more likely to be detected compared with control, and the same strain of bacteria was detected in ascites [88,89]. Therefore, bacterial translocation is considered to be an etiology of the early stage of SBP. Sarcopenia could worsen as liver disease progresses. Hanai et al. reported that in patients with Child–Pugh class A, B, and C, the rate of decrease of skeletal muscle per year was 1.3%, 3.5%, and 6.1% [90]. Cirrhosis to dysbiosis ratio (CDR, described later) decreases with the worsening of liver function [91,92]. Considering this evidence, the severity of sarcopenia in LC can be closely associated with the severity of dysbiosis.

Dysbiosis in LC can cause: (1) decreased bacterial diversity [91], (2) decreased SCFA (energy source in human body) production [93], (3) collapse of TJ and subsequent increased intestinal permeability (leaky gut syndrome) [94], (4) antioxidant dysfunction [95], and (5) endotoxemia [96,97]. These can be associated with anabolic resistance, chronic inflammation, mitochondrial dysfunction, oxidative stress, and insulin resistance, which can lead to LC progression and subsequent development of sarcopenia in LC patients [25].

## 5. Surrogate Markers for the Severity of Dysbiosis in Liver Cirrhosis

The ratio of beneficial to potentially harmful bacterial taxa, or the CDR (autochthonous to non-autochthonous taxa ratio), has been proposed as an index of alterations in the GM [92]. Examples of benign and autochthonous gut taxa include Lachnospiraceae, Ruminococcaceae, Veillonellaceae, and Clostridiales Incertae Sedis XIV, while pathogenic gut taxa include Enterobacteriaceae and Bacteroidaceae [92]. A low CDR may suggest a decrease in beneficial bacteria and/or an excessive abundance of harmful taxa. Altered bacterial function has also been demonstrated in LC patients compared with healthy controls [2,12]. In other words, a deficit of autochthonous non-pathogenic bacteria and an excessive growth of potentially pathogenic bacteria are common characteristics in LC patients [2,12,92]. Progressive alterations in the GM were found in worsening LC, such that the CDR was significantly decreased with liver disease progression [45]. In contrast, CDR and the GM were unchanged in patients without disease progression (i.e., stable liver disease) [92]. Bajaj et al. demonstrated that in hospitalized patients with LC (*n* = 180), dysbiosis of the GM as assessed by CDR, etc., on admission can be associated with elevated risk of extra-hepatic organ failure, ACLF, and mortality, independent of baseline clinical characteristics [98]. Another study reported a significant fungal dysbiosis in LC patients [99]. In their results, the GM in LC patients altered differentially with antibiotics and PPI use, and stool bacterial/fungal profiles predicted 90-day hospitalizations well in LC patients [99].

## 6. Small Intestine Bacterial Overgrowth in Liver Cirrhosis

Small intestine bacterial overgrowth (SIBO) is common in LC patients as a result of intestinal motility disorders and delayed transit times, and exacerbation of LC is associated with SIBO [100]. In a previous study, the multivariate analysis showed that SIBO (HR = 8.10, *p* = 0.002) and ascites (HR = 4.56, *p* = 0.022) were independently associated with the occurrence of malnutrition [100]. The severity of SIBO can be linked to the severity of LC status [101]. Increased intestinal permeability may help bacteria move into the systemic circulation. SIBO has been implicated as an important risk factor in the etiology of both SBP and HE in LC patients [102,103]. Thus, SIBO is deeply involved in the progression of CLD, which may be linked to sarcopenia [102,103].

## 7. Dysbiosis and Bile Acid

Bile acids (BAs) are synthesized from cholesterol in the liver and metabolized by the GM into secondary BAs (e.g., deoxycholic acid (DCA)). There is a positive correlation between abundance of Ruminococcaceae (benign bacteria) and DCA [104]. Most of the BAs that reach the ileum are reabsorbed and repeat gut–liver circulation, but some BAs reach the colon and are converted by the GM (secondary BAs). Secondary BAs regulate functions related to glucose and fat metabolism in the liver [105,106]. In mice with dysbiosis, the expression levels of proteins in the liver involved in glycogen metabolism, cholesterol biosynthesis, and BA biosynthesis were altered, and these changes were recovered by supplementation with secondary BAs [105]. Atrophic change of skeletal muscle was confirmed in rats lacking the BA receptor TGR5 expressed in skeletal muscle, indicating that TGR5 enhances skeletal muscle hypertrophy and skeletal muscle cell differentiation [107]. In LC patients, a decreased conversion of primary to secondary fecal BAs due to dysbiosis can be found [104,108]. Preventive effects of secondary BAs on sarcopenia in LC patients are currently unknown.

## 8. Gut Microbiome in Patients with CLDs and Other Diseases

Inoue et al. demonstrated informative data with regard to dysbiosis in patients with hepatitis C virus (HCV) as summarized below [91]: (1) Even in HCV carriers with normal liver function (persistent normal alanine aminotransferase (PNALT)), alterations in GM already appeared. (2) As the liver function worsened from PNALT or chronic hepatitis to LC or HCC, the proportion of resident bacteria in the GM decreased, the types of bacteria that compose the GM decreased, and the pH of feces increased. These results mean that dysbiosis of the GM was occurring. (3) As the liver function worsened, *Streptococcus salivarius*, which is a genus of streptococci, increased abnormally in the GM. It is possible that these bacteria decomposed urea in the intestinal tract to produce ammonia, and the pH of feces elevated. Avoiding proliferation of such ammonia-producing bacteria may lead to the prevention or treatment of hyperammonemia seen in LC patients. (4) Early interventions for GM (administration of probiotics, administration of appropriate antibiotics, oral care, etc.) may suppress the progression of hepatitis C and the development of HCC [91]. A recent study reported an increase in potentially pathologic bacteria and a decrease in potentially beneficial bacteria or genes in patients with hepatitis B virus, which is similar to data in patients with HCV [109].

In recent years, the association between dysbiosis and alcoholic hepatitis associated with excessive drinking has been receiving more attention [110]. In alcoholic liver injury, intestinal permeability is increased (i.e., leaky gut), and pathogen-associated molecular patterns (PAMPs) represented by endotoxin (lipopolysaccharide (LPS)) derived from bacteria reach the liver through the portal vein and cause liver damage by activating Kupffer cells [110]. Endotoxin is mainly present in the cell wall of Gram-negative bacteria. Intestinal sterilization with antibiotics and probiotics such as lactobacillus can suppress alcoholic liver injury, and in LPS receptor CD14 and toll-like receptor (TLR) 4 knockout mice, the onset of liver injury by chronic alcohol administration is suppressed [111,112]. In addition, it has been suggested that the onset and progression of non-alcoholic steatohepatitis (NASH) are associated with intestinal endotoxin [113]. In patients with NAFLD, low dose endotoxin can overreact to cause NASH [113].

GM can be affected by aging. Odamaki et al. demonstrated using fecal samples from 367 healthy Japanese persons between 0 and 104 years that certain transition types of GM were enriched in infants (e.g., Bifidobacterium), adults (e.g., Lachnospiraceae), elderly individuals (e.g., Bacteroides), and both infant and elderly subjects (e.g., Enterobacteriaceae) [114]. On the other hand, Flemer et al. reported in their prospective study that the GM in CRC patients differs significantly from that of healthy persons throughout the colon [115]. Coronavirus disease-19 (COVID-19) has been rapidly becoming a global challenge. A recent study reported that in patients with COVID-19, fecal GM alterations were associated with COVID-19 severity [116]. Additionally, COVID-19 is likely to be accompanied by liver damage, and caution is required especially in LC patients for the disease progression caused by COVID-19 [117]. COVID-19 patients with liver disease had significantly higher mortality rates than those without (HR = 3.0, *p* = 0.001) [117]. Interestingly, Ren et al. reported that GM markers validated strong diagnosis potential for the early stage of HCC (area under the receiver operating characteristic curve = 0.8064) [118].

## 9. Antibiotics, Dysbiosis, Ammonia-Lowering Strategies, and Sarcopenia

One possible factor which alters GM is the taking of antibiotics. While antibiotics are effective for the treatment and prevention of bacterial infections, they can cause dysbiosis [119]. Rifaximin, which is a poorly-absorbable rifamycin-based antibiotic, acts on GM that are a source of ammonia to reduce ammonia production, thereby improving hyperammonemia in HE [120,121]. Rifaximin has an effect of inhibiting bacterial RNA synthesis, and the antibacterial activity of rifaximin covers a broad spectrum of bacteria [120,121]. Rifaximin has a favorable safety profile for long-term administration compared with oral systemic antibiotics [121]. Kaji et al. demonstrated in their 20 decompensated LC patients that 4 weeks rifaximin therapy improved hyperammonemia and reduced endotoxin activity in direct correlation with the decline in serum ammonia levels, without impact on the composition of GM [97]. Rifaximin also acts favorably on the serum pro-inflammatory cytokine profile and fecal secondary BA levels [122,123]. Rifaximin seems to alter the secondary to primary BA ratio in compensated LC patients, which can be associated with reduction in endotoxemia and reduction in harmful metabolite levels [104]. In addition, rifaximin appears to be effective and safe for the treatment of SIBO [124]. The clinical activity of rifaximin may be attributed to the effects on metabolic function of the GM, rather than an alteration in relative bacterial abundance [125].

A recent meta-analysis (5 studies, comprising 555 patients) reported that rifaximin therapy may be effective in preventing SBP in patients with LC and ascites compared with systemic absorbed antibiotics and compared with placebo [126]. Flamm et al. demonstrated that in patients with Model for End-stage Liver Disease score 12 or greater and international normalized ratio 1.2 or greater, rifaximin group (*n* = 140) reduced the relative risk of any first complication (HE, SBP, variceal bleeding, acute kidney injury, or hepatorenal syndrome) experienced during the study period by 59% [HR = 0.41, 95% CI = 0.25–0.67; *p* < 0.001] vs. placebo group (*n* = 159) [127]. Kumar et al. reported that in rats with port-systemic shunts, the increase in skeletal muscle myostatin expression, suppressed mTORC1 function, and hyperammonemia-related stress response (i.e., autophagy markers) were reversed by ammonia-lowering therapy, concluding that it can lead to the improvement in skeletal muscle phenotype and function [128]. However, the preventive effects of rifaximin on sarcopenia incidence or progression in LC patients remain unclear. Table 2 demonstrates randomized controlled trials (RCTs) published since 2010 regarding the effects of rifaximin on outcomes in patients with decompensated LC [129,130,131,132,133,134,135,136,137,138,139,140,141,142,143,144,145,146,147,148,149,150,151,152,153]. RCTs with the improvement of sarcopenia as a primary endpoint are not found. In our hypothesis, rifaximin treatment in LC patients with sarcopenia potentially has an impact on the improvement of sarcopenia through the improvement of hyperammonemia and subsequent hypermyostatinemia in skeletal muscles. Further exams with regard to the effect of rifaximin on sarcopenia in LC patients will be required to confirm these results. l-carnitine therapy, which is also an ammonia-lowering therapy, can improve sarcopenia in LC patients [154,155].

## 10. Probiotics, Dysbiosis, Ammonia-Lowering Strategies, and Sarcopenia

Probiotics are defined as microorganisms that have positive effects on the human body, or drugs and foods containing them. Probiotics may act on the GM, intestinal epithelial cells, immunocompetent cells present in the intestinal mucosa, etc. [85,156]. Many neurotoxic substances are derived from the GM, and the usefulness of probiotics for improving the composition of GM has been investigated as a treatment for HE [85,156]. Probiotics enhance the expression of TJ-related proteins with improvement of dysbiosis and improve the intestinal barrier function [157,158].

A previous meta-analysis demonstrated that probiotics reduce the risk of hospitalization and the progression to overt HE to the same extent as lactulose in patients with minimal HE, but do not affect mortality [125]. On the other hand, another systematic review comparing probiotics with placebo or no treatment summarized as follows: (1) There was no significant difference in mortality from any cause. (2) The non-recovery rate and the incidence of adverse events including HE were lower in the probiotics group, but the effect on hospitalization was unclear. (3) Quality of life was slightly improved in the probiotics group. (4) In comparison of probiotics and lactulose, the effects on mortality rate from any cause, non-recovery rate, incidence of adverse events including HE, hospitalization, and quality of life were unable to be assessed due to the low quality of evidence [159]. Table 3 demonstrates RCTs published since 2010 regarding the effects of probiotics on outcomes in patients with decompensated LC [160,161,162,163,164,165,166,167,168,169,170,171,172]. RCTs with the improvement of sarcopenia as a primary endpoint are not found. The effects of probiotics on sarcopenia in LC patients are unclear as well as those of rifaximin. In mice given probiotics (*Lactobacillus paracasei* PS23), aging-related muscle mass decline and muscle strength decline significantly improved [173].

## 11. Exercise and Gut Microbiota in Liver Cirrhosis

Across a range of chronic diseases, several guidelines recommend exercise training. It has been found that exercise can change the composition of the GM, which leads to an intestinal flora with a beneficial metabolic system [174]. Clarke et al. showed that the GM of rugby players was highly diverse, clearly different from normal healthy subjects, and that there was a high positive correlation between the diversity of GM and protein intake [175]. Athletes presented a higher level of SCFA-producing bacteria and bacterial genes related to nutritional metabolism compared with sedentary controls [176]. Aerobic and resistance training revealed improved GM composition and functionality in rats with NAFLD [174]. However, only a few reports have shown exercise-related alterations on the GM in humans, and most evidence comes from non-randomized studies. Huber et al. reported that in 41 NAFLD patients receiving an 8-week exercise program, increased metagenomic richness of the GM (i.e., increased diversity) was observed [177]. To date, there are no clinical studies or RCTs looking specifically at exercise and the GM in patients with LC with sarcopenia, although exercise can decrease HVPG in LC patients [178]. If the improvement of the GM in LC patients with sarcopenia by exercise is confirmed, future treatment strategies for LC patients with sarcopenia and dysbiosis will be changed.

## 12. Closing Remarks

**Figure 2:**
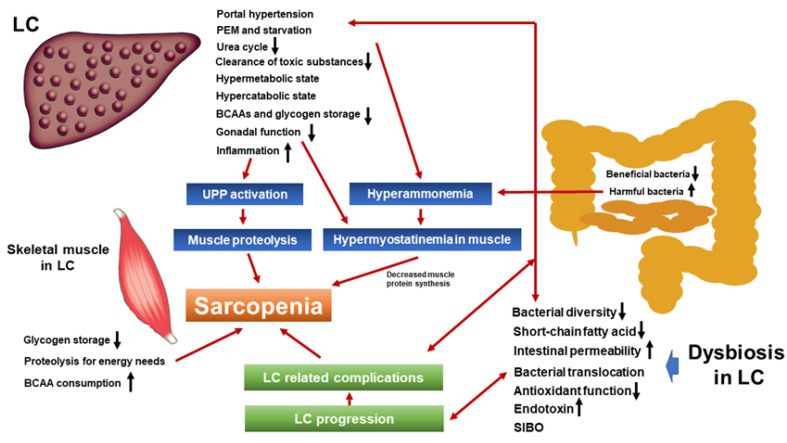
Schematic explanation of the relationship between sarcopenia and dysbiosis in patients with liver cirrhosis. LC: liver cirrhosis; PEM: protein-energy malnutrition; BCAA: branched-chain amino acid; UPP: ubiquitin-proteasome pathway; SIBO: small intestine bacterial overgrowth.

Interactions between dietary nutrients and GM promote host nutrition and health via various signaling pathways, and to maintain and promote human health, beneficial bacteria should be dominant in the GM [7]. In LC patients, these interactions can be disturbed due to PEM, amino acid imbalance, dysbiosis, etc., which can cause sarcopenia. Sarcopenia is a public health problem that cannot be overlooked. As mentioned earlier, skeletal muscle is considered to be a metabolic organ. When understanding the pathophysiology of LC, we must always keep in mind the relationship between organs including skeletal muscles and the digestive tract, that is, the organ network. In this article, we overviewed the current knowledge of the relationship between dysbiosis and sarcopenia in patients with LC. A summarized scheme is shown in Figure 2. In the past decade, marked advances have been made in this research field. Our current research questions are whether or not rifaximin, probiotics, or exercise training can improve sarcopenia in LC through the improvement of the GM. To the best of our knowledge, appropriate RCTs to address these research questions cannot be found. Future research is eagerly awaited.

## Figures and Tables

**Table 1:** Three types of intestinal barrier.

**1. Environmental Barrier**	Gut microbiota
**2. Biological Barrier**	Antimicrobial peptide
	Immune cells
**3. Physical Barrier**	Mucus layer
	Tight junction

**Table 2:** Randomized controlled trials published since 2010 regarding the effect of rifaximin in patients with decompensated liver cirrhosis. MHE: minimal hepatic encephalopathy; BT: bacterial translocation; OHE: overt hepatic encephalopathy; sMR: soluble mannose receptor; HRS: hepatorenal syndrome.

Author (Year)	Treatment	Design	Target Patients	*n*	Primary Endpoint	Main Result
Schulz C, et al. (2019) [129]	Rifaximin 550 mg twice daily alone continuously for 3 months vs. rifaximin combined with lactulose 30–60 mL daily for 3 months	RCT	Decompensated LC with MHE	5	MHE improvement	Significant improvement of MHE in all patients. No statistically significant changes in the bacterial community profile at each time point.
Kimer N, et al. (2018) [130]	Rifaximin for 4 weeks vs. placebo	RCT	Decompensated LC	54	BT and inflammation	No impact on the inflammatory state and only minor effects on BT and intestinal bacterial composition
Nutt NI, et al. (2018) [131]	Lactulose vs. Lactulose+ rifaximin 550 mg twice daily	RCT	HE due to decompensated LC	130	HE	No significant difference on HE (*p* = 0.276).
Mekky MA, et al. (2018) [132]	Rifaximin vs. metronidazole	RCT	Decompensated LC with an acute episode of OHE	120	OHE improvement	OHE improvement: 46 patients (76.7%) in the metronidazole group vs. 45 (75%) in the rifaximin group (*p* = 0.412).
Higuera-de-la-Tijera F, et al. (2018) [133]	Lactulose vs. L-ornithine L-aspartate (LOLA) vs. rifaximin vs. placebo	RCT	Decompensated LC with variceal bleeding	87	HE development	Lactulose vs. placebo: 54.5% vs. 27.3%, *p* = 0.06 LOLA vs. placebo: 54.5% vs. 22.7%, *p* = 0.03 Rifaximin vs. placebo: 54.5% vs. 23.8%, *p* = 0.04.
Kimer N, et al. (2018) [134]	Rifaximin for 4 weeks vs. placebo	RCT	Decompensated LC	54	Macrophage markers s CD163, sMR	sCD163 and sMR were associated with liver disease severity. No effect of rifaximin on sCD163 and sMR.
Goyal O, et al. (2017) [135]	Rifaximin (1200 mg/day) vs. lactulose (30–120 mL/day) for 3 months	RCT	Decompensated LC with MHE	112	MHE reversal	MHE reversal at 3 months: 73.7% (42/57) in the rifaximin group and 69.1% (38/55) in the lactulose group (*p* = 0.677).
Lauridsen MM, et al. (2017) [136]	Lactulose plus BCAAs plus rifaximin vs. triple placebos for 3 months	RCT	Decompensated LC without clinically manifest HE	44	Continuous reaction test time (CRT)	ΔCRT: 0.50 ± 0.20 vs. 0.13 ± 0.12 (*p* = 0.06).
Lim YL, et al. (2017) [137]	Propranolol monotherapy vs. rifaximin and propranolol combination therapy	RCT	Decompensated LC	64	HVPG	HVPG response rates: 56.2% in the propranolol vs. 87.5% in the combination, (*p* = 0.034).
Ibrahim ES, et al. (2017) [138]	Rifaximin 550 mg twice daily for 12 weeks vs. placebo	RCT	Decompensated LC	80	HRS occurrence	HRS occurrence: 9 (22.5%) in the control group vs. 2 (5%) in the rifaximin group; *p* = 0.048.
Kimer N, et al. (2017) [139]	Rifaximin for 4 weeks vs. placebo	RCT	Decompensated LC	54	HVPG	No significant difference on HVPG (*p* = 0.94).
Elfert A, et al. (2016) [140]	Rifaximin 1200 mg daily vs. norfloxacin 400 mg daily for 6 months	RCT	Decompensated LC with a previous episode of SBP	262	Prevention of SBP	Recurrence rate of SBP: 3.88% in the rifaximin vs. 14.13% in the norfloxacin (*p* = 0.04) Mortality: 13.74% in the rifaximin vs. 24.43% in the norfloxacin (*p* = 0.044).
Sidhu, et al. (2016) [141]	Rifaximin 400 mg thrice a day vs. lactulose 30–120 mL/day	RCT	MHE due to decompensated LC	112	MHE improvement	MHE reversal at 3 months: 73.7% (42/57) in the rifaximin arm and 69.1% (38/55) in the lactulose arm (*p* > 0.05).
Assem M, et al. (2016) [142]	Alternating use of norfloxacin and rifaximin vs. norfloxacin or rifaximin alone	RCT	Decompensated LC	334	Primary prophylaxis of SBP	Primary prophylaxis of SBP: 74.7% vs. 56.4% vs. 68.3%, (*p* < 0.048).
Zeng X, et al. (2015) [143]	Low dose rifaximin (800 mg/day, 2 weeks) vs. high dose rifaximin (1200 mg/day, 2 weeks) vs. placebo	RCT	Decompensated LC	43	Endotoxemia	1.1 ± 0.8 EU/mL in the low dose rifaximin (*p* < 0.05) 1.0 ± 0.8 EU/mL in the high dose rifaximin (*p* < 0.05) 2.5 ± 1.8 EU/mL in the control group.
Mostafa T, et al. (2015) [144]	Rifaximin vs. norfloxacin for 6 months	RCT	Decompensated LC	70	Inflammatory markers	No significant difference on TNF-α, IL-6, and IL-10.
Khokhar N, et al. (2015) [145]	Rifaximin 550 mg once a day vs. rifaximin 550 mg twice daily	RCT	Decompensated LC with at least one episode of HE	306	HE recurrence	Twenty-seven patients in rifaximin 550 mg once a day and 54 patients in rifaximin 550 mg twice daily with breakthrough episode of HE (*p* = 0.088).
Sharma K, et al. (2014) [146]	L-ornithine l-aspartate (LOLA) vs. rifaximin vs. probiotics vs. placebo for 2 months	RCT	Decompensated LC with MHE	124	MHE improvement	Critical flicker frequency scores and improvement in psychometric tests after treatment were significantly higher (*p* < 0.05) for LOLA, rifaximin, and probiotics as compared with placebo group.
Ali B, et al. (2014) [147]	Rifaximin 550 mg twice daily for 6 months vs. placebo	RCT	Decompensated LC with at least one episode of HE	126	HE recurrence	Free of hepatic encephalopathy during study period: 40 out of 63 patients in the placebo group and 35 patients out of 63 patients in the rifaximin group (*p* = 0.56).
Sharma BC, et al. (2013) [148]	Lactulose plus rifaximin 1200 mg/day vs. lactulose plus placebo	RCT	Decompensated LC with OHE	120	Complete reversal of HE	Forty-eight (76%) in lactulose plus rifaximin compared with 29 (50.8%) in lactulose plus placebo had complete reversal of HE (*p* < 0.004).
Kalambokis GN, et al. (2012) [149]	Rifaximin 1200 mg/day vs. no treatment	RCT	Alcoholic LC with thrombocytopenia	23	Thrombocytopenia	In the rifaximin group, platelet counts increased significantly (83,100 ± 9700 vs. 99,600 ± 11,200/μL; *p* = 0.006) with significant reductions in endotoxin (1.28 ± 0.41 vs. 2.54 ± 0.86 EU/mL; *p* = 0.005).
Sidhu SS, et al. (2011) [150]	Placebo vs. rifaximin (1200 mg/day) for 8 weeks	RCT	Decompensated LC with MHE	94	MHE improvement	Significantly more patients in the rifaximin group presented reversal of MHE (75.5% (37/49) vs. 20% (9/45) in the placebo group; *p* < 0.0001).
Bajaj JS, et al. (2011) [151]	Rifaximin 550 mg twice daily vs. placebo for 8 weeks	RCT	Decompensated LC with MHE and current drivers	42	Improvement in driving performance	Rifaximin group made significantly greater improvements than placebo group in avoiding total driving errors (76% vs. 31%; *p* = 0.013), speeding (81% vs. 33%; *p* = 0.005), and illegal turns (62% vs. 19%; *p* = 0.01).
Sanyal A, et al. (2011) [152]	Rifaximin 550 mg twice daily vs. placebo for 6 months	RCT	Decompensated LC with a documented history of recurrent HE	219	Chronic Liver Disease Questionnaire (CLDQ) score	The time-weighted averages of the overall CLDQ score and each domain score were significantly higher in the rifaximin group vs. placebo (*p*-values ranged from 0.0087 to 0.0436).
Bass NM, et al. (2010) [153]	Rifaximin 550 mg twice daily vs. placebo for 6 months	RCT	Decompensated LC with remission from HE	299	First breakthrough episode of HE	Rifaximin significantly reduced the risk of an episode of HE compared with placebo over 6 months (HR with rifaximin, 0.42; 95% CI, 0.28 to 0.64; *p* < 0.001).

**Table 3:** Randomized controlled trials published since 2010 regarding the effect of probiotics in patients with decompensated liver cirrhosis. MHE: minimal hepatic encephalopathy; OHE: overt hepatic encephalopathy.

Author (Year)	Treatment	Design	Target Patients	*n*	Primary Endpoint	Main Result
Xia X, et al. (2018) [160]	Probiotics (Clostridium butyricum combined with B. infantis) vs. no probiotics for 3 months	RCT	Decompensated HBV-LC without OHE	67	Cognitive function and quantitative assessment of predominant fecal bacteria	The cognition was significantly improved after probiotic treatment. The predominant bacteria (Clostridium cluster I and Bifidobacterium) were significantly enriched in the probiotics-treated group.
Horvath A, et al. (2016) [161]	Probiotics (eight different bacterial strains) vs. placebo for 6 months	RCT	Decompensated LC	80	The change in phagocytic capacity of neutrophils	A significant increase in neutrophil resting burst (2.6–3.2%, *p* = 0.0134) and neopterin levels (7.7–8.4 nmol/L, *p* = 0.001) in the probiotics group but not in the placebo group.
Dhiman RK, et al. (2014) [162]	A probiotic preparation (VSL#3, 9 × 10(11) bacteria) vs. placebo for 6 months	RCT	Decompensated LC who had recovered from an episode of HE	130	Development of breakthrough HE	Development of breakthrough HE: 34.8% in the probiotic group vs. 51.6% in the placebo group; HR, 0.65; 95% CI, 0.38-1.11; *p* = 0.12.
Lunia MK, et al. (2014) [163]	Probiotics (1 × 10(8) colony-forming units, 3 times daily) vs. control	RCT	Decompensated LC without OHE	160	The development of OHE	Seven subjects in the probiotics group and 14 controls developed OHE (*p* < 0.05; HR for controls vs. probiotic group, 2.1; 95% CI, 1.31–6.53).
Bajaj JS, et al. (2014) [164]	Probiotic Lactobacillus GG (LGG) vs. placebo for 8 weeks	RCT	Decompensated LC	37	Endotoxin, systemic inflammation and microbiome	Only in the LGG group, endotoxemia and TNF-α decreased, microbiome changed (reduced Enterobacteriaceae and increased Clostridiales Incertae Sedis XIV and Lachnospiraceae relative abundance).
Gupta N, et al. (2013) [165]	Propranolol plus placebo vs. propranolol plus antibiotics (norfloxacin 400 mg twice daily) vs. propranolol plus probiotic (VSL#3, 900 billion/day)	RCT	Decompensated LC with large esophageal varices without history of variceal bleeding	94	HVPG	The mean fall in HVPG was greater with either adjunctive probiotics (3.7 mmHg vs. 2.1 mmHg, *p* = 0.061) or adjunctive antibiotics (3.4 mmHg) than with propranolol alone.
Jayakumar S, et al. (2013) [166]	Probiotics (VSL#3) vs. placebo for 2 months	RCT	Decompensated LC with an HVPG 10 mmHg or more	17	HVPG	Median HVPG change from baseline -11.6% in the probiotics vs. +2.8% in the placebo (*p* > 0.05)
Agrawal A, et al. (2012) [167]	Lactulose vs. three capsules of probiotics vs. no therapy	RCT	Decompensated LC who had recoverd from HE	235	The development of OHE	The development of OHE: lactulose vs. probiotics, *p* = 0.349; probiotics vs. no therapy, *p* = 0.02; lactulose vs. no therapy, *p* = 0.001).
Pande C, et al. (2012) [168]	Norfloxacin 400 mg/day with probiotics capsules vs. norfloxacin with a placebo for 6 months	RCT	Decompensated LC who had either recovered from SBP or who were at a high risk for SBP	110	The occurrence of SBP	The frequencies of SBP were similar in the two groups. The cumulative probability of mortality was also similar.
Pereg D, et al. (2011) [169]	Probiotics vs. placebo for 6 months	RCT	Decompensated LC with at least one major complication of LC in the past	36	The effect on clinical and laboratory parameters	Probiotics was not associated with significant differences in either clinical or laboratory parameters between the two groups.
Mittal VV, et al. (2011) [170]	Lactulose vs. probiotics vs. L-ornithine L-aspartate (LOLA) vs. no therapy for 3 months	RCT	Decompensated LC with MHE	322	The improvement of MHE	The improvement of MHE: lactulose, 47.5%; probiotics, 35%; LOLA, 35%; no therapy, 10%. MHE improved significantly in all three treatment groups compared with no treatment (*p* = 0.006).
Saji S, et al. (2011) [171]	Probiotics vs. placebo	RCT	Decompensated LC with MHE	43	The improvement of MHE	There was no statistically significant change in the parameters (arterial ammonia, evoked responses and number connection test) between probiotics and placebo.
Malaguarnera M, et al. (2010) [172]	Bifidobacterium plus fructo-oligosaccharides (FOS) vs. lactulose for 2 months	RCT	Decompensated LC with HE	125	The improvement of HE	Bifidobacterium plus FOS-treated group compared with lactulose group showed a significant decrease of serum ammonia (*p* < 0.001), Trail Making Test A (*p* < 0.05) and B (*p* < 0.001), and a significant increase of Symbol Digit Modalities Test (*p* < 0.001) and Block Design Test (*p* < 0.001).

## Abbreviations

GM	gut microbiota
CLD	chronic liver disease
LC	liver cirrhosis
PEM	protein-energy-malnutrition
BCAA	branched-chain amino acid
HCC	hepatocellular carcinoma
SBP	spontaneous bacterial peritonitis
HE	hepatic encephalopathy
ACLF	acute on chronic liver failure
JSH	Japanese Society of Hepatology
CT	computed tomography
HCV	hepatitis C virus
PNALT	persistent normal alanine aminotransferase
COVID-19	Coronavirus disease-19
PAMPs	pathogen-associated molecular patterns
LPS	lipopolysaccharide
TLR	toll like receptor
NASH	non-alcoholic steatohepatitis
NAFLD	non-alcoholic fatty liver disease
HR	hazard ratio
CI	confidence interval
PPI	proton pump inhibitor
mTORC1	mammalian target of rapamycin complex1
TNF-α	tumor necrosis factor-alpha
CRC	colorectal cancer
SCFAs	short-chain fatty acids
HVPG	hepatic venous pressure gradient
TJ	tight junction
SIBO	small intestine bacterial overgrowth
BAs	bile acids
DCA	deoxycholic acid
RCT	randomized controlled trial

## References

[1] Tripathi A., Debelius J., Brenner D.A., Karin M., Loomba R., Schnabl B., Knight R. (2018). The Gut-Liver Axis and the Intersection With the Microbiome. Nat. Rev. Gastroenterol. Hepatol..

[2] Milosevic I., Vujovic A., Barac A., Djelic M., Korac M., Radovanovic Spurnic A., Gmizic I., Stevanovic O., Djordjevic V., Lekic N. (2019). Gut-Liver Axis, Gut Microbiota, and Its Modulation in the Management of Liver Diseases: A Review of the Literature. Int. J. Mol. Sci..

[3] Cazorla S.I., Maldonado-Galdeano C., Weill R., De Paula J., Perdigón G.D.V. (2018). Oral Administration of Probiotics Increases Paneth Cells and Intestinal Antimicrobial Activity. Front. Microbiol..

[4] Albillos A., de Gottardi A., Rescigno M. (2020). The Gut-Liver Axis in Liver Disease: Pathophysiological Basis for Therapy. J. Hepatol..

[5] Solga S.F., Diehl A.M. (2004). Gut Flora-Based Therapy in Liver Disease? The Liver Cares About the Gut. Hepatology.

[6] Andoh A. (2016). Physiological Role of Gut Microbiota for Maintaining Human Health. Digestion.

[7] Belizário J.E., Faintuch J., Garay-Malpartida M. (2018). Gut Microbiome Dysbiosis and Immunometabolism: New Frontiers for Treatment of Metabolic Diseases. Mediat. Inflamm..

[8] Tilg H., Cani P.D., Mayer E.A. (2016). Gut Microbiome and Liver Diseases. Gut.

[9] Federico A., Dallio M., Caprio G.G., Ormando V.M., Loguercio C. (2017). Gut Microbiota and the Liver. Minerva Gastroenterol. Dietol..

[10] Acharya C., Sahingur S.E., Bajaj J.S. (2017). Microbiota, Cirrhosis, and the Emerging Oral-Gut-Liver Axis. JCI Insight.

[11] Woodhouse C.A., Patel V.C., Singanayagam A., Shawcross D.L. (2018). The Gut Microbiome as a Therapeutic Target in the Pathogenesis and Treatment of Chronic Liver Disease. Aliment. Pharmacol. Ther..

[12] Qin N., Yang F., Li A., Prifti E., Chen Y., Shao L., Guo J., Le Chatelier E., Yao J., Wu L. (2014). Alterations of the Human Gut Microbiome in Liver Cirrhosis. Nature.

[13] Amrane S., Hocquart M., Afouda P., Kuete E., Pham T.P., Dione N., Ngom I.I., Valles C., Bachar D., Raoult D. (2019). Metagenomic and Culturomic Analysis of Gut Microbiota Dysbiosis During Clostridium Difficile Infection. Sci. Rep..

[14] Halfvarson J., Brislawn C.J., Lamendella R., Vázquez-Baeza Y., Walters W.A., Bramer L.M., D’Amato M., Bonfiglio F., McDonald D., Gonzalez A. (2017). Dynamics of the Human Gut Microbiome in Inflammatory Bowel Disease. Nat. Microbiol..

[15] Shen F., Zheng R.D., Sun X.Q., Ding W.J., Wang X.Y., Fan J.G. (2017). Gut Microbiota Dysbiosis in Patients With Non-Alcoholic Fatty Liver Disease. Hepatobiliary Pancreat. Dis. Int..

[16] Fazlollahi M., Chun Y., Grishin A., Wood R.A., Burks A.W., Dawson P., Jones S.M., Leung D.Y.M., Sampson H.A., Sicherer S.H. (2018). Early-life Gut Microbiome and Egg Allergy. Allergy.

[17] Dzidic M., Abrahamsson T.R., Artacho A., Björkstén B., Collado M.C., Mira A., Jenmalm M.C. (2017). Aberrant IgA Responses to the Gut Microbiota During Infancy Precede Asthma and Allergy Development. J. Allergy Clin. Immunol..

[18] Kang D.W., Adams J.B., Gregory A.C., Borody T., Chittick L., Fasano A., Khoruts A., Geis E., Maldonado J., McDonough-Means S. (2017). Microbiota Transfer Therapy Alters Gut Ecosystem and Improves Gastrointestinal and Autism Symptoms: An Open-Label Study. Microbiome.

[19] Teng F., Klinger C.N., Felix K.M., Bradley C.P., Wu E., Tran N.L., Umesaki Y., Wu H.J. (2016). Gut Microbiota Drive Autoimmune Arthritis by Promoting Differentiation and Migration of Peyer’s Patch T Follicular Helper Cells. Immunity.

[20] Lai J.C., Covinsky K.E., Dodge J.L., Boscardin W.J., Segev D.L., Roberts J.P., Feng S. (2017). Development of a Novel Frailty Index to Predict Mortality in Patients With End-Stage Liver Disease. Hepatology.

[21] Nishikawa H., Enomoto H., Yoh K., Iwata Y., Sakai Y., Kishino K., Ikeda N., Takashima T., Aizawa N., Takata R. (2019). Combined Albumin-Bilirubin Grade and Skeletal Muscle Mass as a Predictor in Liver Cirrhosis. J. Clin. Med..

[22] Nishikawa H., Enomoto H., Yoh K., Iwata Y., Sakai Y., Kishino K., Ikeda N., Takashima T., Aizawa N., Takata R. (2018). Health-Related Quality of Life in Chronic Liver Diseases: A Strong Impact of Hand Grip Strength. J. Clin. Med..

[23] Müller M.J., Loyal S., Schwarze M., Lobers J., Selberg O., Ringe B., Pichlmayr R. (1994). Resting Energy Expenditure and Nutritional State in Patients With Liver Cirrhosis before and after Liver Transplantation. Clin. Nutr..

[24] Lai J.C., Rahimi R.S., Verna E.C., Kappus M.R., Dunn M.A., McAdams-DeMarco M., Haugen C.E., Volk M.L., Duarte-Rojo A., Ganger D.R. (2019). Frailty Associated With Waitlist Mortality Independent of Ascites and Hepatic Encephalopathy in a Multicenter Study. Gastroenterology.

[25] Ebadi M., Bhanji R.A., Mazurak V.C., Montano-Loza A.J. (2019). Sarcopenia in cirrhosis: From pathogenesis to interventions. J. Gastroenterol..

[26] Poh H.O., Amber H., Vera C.M., Khaled D., Ravi B., Susan M.G., Diana R.M. (2019). Sarcopenia in Chronic Liver Disease: Impact on Outcomes. Liver Transpl..

[27] Bhanji R.A., Moctezuma-Velazquez C., Duarte-Rojo A., Ebadi M., Ghosh S., Rose C., Montano-Loza A.J. (2018). Myosteatosis and Sarcopenia Are Associated With Hepatic Encephalopathy in Patients with Cirrhosis. Hepatol. Int..

[28] Hanai T., Shiraki M., Imai K., Suetsugu A., Takai K., Moriwaki H., Masahito S. (2019). Reduced handgrip strength is predictive of poor survival among patients with liver cirrhosis: A sex-stratified analysis. Hepatol. Res..

[29] Nishikawa H., Enomoto H., Ishii A., Iwata Y., Miyamoto Y., Ishii N., Yuri Y., Takata R., Hasegawa K., Nakano C. (2017). Prognostic significance of low skeletal muscle mass compared with protein-energy malnutrition in liver cirrhosis. Hepatol. Res..

[30] Bunchorntavakul C., Reddy K.R. (2020). Review article: Malnutrition/sarcopenia and frailty in patients with cirrhosis. Aliment. Pharmacol. Ther..

[31] Schneeweiss B., Graninger W., Ferenci P., Eichinger S., Grimm G., Schneider B., Laggner A.N., Lenz K., Kleinberger G. (1990). Energy metabolism in patients with acute and chronic liver disease. Hepatology.

[32] Nishikawa H., Osaki Y. (2014). Clinical Significance of Therapy Using Branched-Chain Amino Acid Granules in Patients with Liver Cirrhosis and Hepatocellular Carcinoma. Hepatol. Res..

[33] Nardelli S., Gioia S., Faccioli J., Riggio O., Ridola L. (2019). Sarcopenia and cognitive impairment in liver cirrhosis: A viewpoint on the clinical impact of minimal hepatic encephalopathy. World J. Gastroenterol..

[34] Hiraoka A., Michitaka K., Kiguchi D., Izumoto H., Ueki H., Kaneto M., Kitahata S., Aibiki T., Okudaira T., Tomida H. (2017). Efficacy of Branched-Chain Amino Acid Supplementation and Walking Exercise for Preventing Sarcopenia in Patients with Liver Cirrhosis. Eur. J. Gastroenterol. Hepatol..

[35] Kitajima Y., Takahashi H., Akiyama T., Murayama K., Iwane S., Kuwashiro T., Tanaka K., Kawazoe S., Ono N., Eguchi T. (2018). Supplementation with branched-chain amino acids ameliorates hypoalbuminemia, prevents sarcopenia, and reduces fat accumulation in the skeletal muscles of patients with liver cirrhosis. J. Gastroenterol..

[36] Namba M., Hiramatsu A., Aikata H., Kodama K., Uchikawa S., Ohya K., Morio K., Fujino H., Nakahara T., Murakami E. (2020). Management of refractory ascites attenuates muscle mass reduction and improves survival in patients with decompensated cirrhosis. J. Gastroenterol..

[37] Hiraoka A., Aibiki T., Okudaira T., Toshimori A., Kawamura T., Nakahara H., Suga Y., Azemoto N., Miyata H., Miyamoto Y. (2015). Muscle atrophy as pre-sarcopenia in Japanese patients with chronic liver disease: Computed tomography is useful for evaluation. J. Gastroenterol..

[38] Maurice J., Pinzani M. (2020). The stratification of cirrhosis. Hepatol. Res..

[39] Nagamatsu A., Kawaguchi T., Hirota K., Koya S., Tomita M., Hashida R., Kida Y., Narao H., Manako Y., Tanaka D. (2019). Slow walking speed overlapped with low handgrip strength in chronic liver disease patients with hepatocellular carcinoma. Hepatol. Res..

[40] Nishikawa H., Shiraki M., Hiramatsu A., Moriya K., Hino K., Nishiguchi S. (2016). Japan Society of Hepatology guidelines for sarcopenia in liver disease (1st edition): Recommendation from the working group for creation of sarcopenia assessment criteria. Hepatol. Res..

[41] Arai H., Akishita M., Chen L.K. (2014). Growing research on sarcopenia in Asia. Geriatr. Gerontol. Int..

[42] Alfonso J.C., Gülistan B., Jürgen B., Yves B., Olivier B., Tommy C., Cyrus C., Francesco L., Yves R., Avan A.S. (2019). Writing Group for the European Working Group on Sarcopenia in Older People 2 (EWGSOP2), and the Extended Group for EWGSOP2. Sarcopenia: Revised European consensus on definition and diagnosis. Age Ageing..

[43] Lai J.C., Covinsky K.E., McCulloch C.E., Feng S. (2018). The Liver Frailty Index Improves Mortality Prediction of the Subjective Clinician Assessment in Patients with Cirrhosis. Am. J. Gastroenterol..

[44] Bhanji R.A., Montano-Loza A.J., Watt K.D. (2019). SARCOPENIA IN CIRRHOSIS: Looking beyond the skeletal muscle loss to see the systemic disease. Hepatology.

[45] Fukui H. (2019). Role of Gut Dysbiosis in Liver Diseases: What Have We Learned So Far?. Diseases.

[46] Aquilio E., Spagnoli R., Riggio D., Seri S. (1993). Effects of Zinc on Hepatic Ornithine Transcarbamylase (OTC) Activity. J. Trace. Elem. Electrolytes. Health Dis..

[47] Gangarao D., Dawid K., Bo-Jhih G., Avinash K., Samjhana T., Dharmvir S., Maria H., Srinivasan D. (2016). Metabolic adaptation of skeletal muscle to hyperammonemia drives the beneficial effects of l-leucine in cirrhosis. J. Hepatol..

[48] Katsanos C.S., Kobayashi H., Sheffield-Moore M., Aarsland A., Wolfe R.R. (2006). A high proportion of leucine is required for optimal stimulation of the rate of muscle protein synthesis by essential amino acids in the elderly. Am. J. Physiol. Endocrinol. Metab..

[49] Lin S.Y., Wang Y.Y., Chuang Y.H., Chen C.J. (2016). Skeletal Muscle Proteolysis Is Associated With Sympathetic Activation and TNF-α-ubiquitin-proteasome Pathway in Liver Cirrhotic Rats. J. Gastroenterol. Hepatol..

[50] Lin S.Y., Chen W.Y., Lee F.Y., Huang C.J., Sheu W.H. (2005). Activation of Ubiquitin-Proteasome Pathway Is Involved in Skeletal Muscle Wasting in a Rat Model With Biliary Cirrhosis: Potential Role of TNF-alpha. Am. J. Physiol. Endocrinol. Metab..

[51] Beyer I., Mets T., Bautmans I. (2012). Chronic low-grade inflammation and age-related sarcopenia. Curr. Opin. Clin. Nutr. Metab. Care.

[52] Milan G., Romanello V., Pescatore F., Armani A., Paik J.H., Frasson L., Seydel A., Zhao J., Abraham R., Goldberg A.L. (2015). Regulation of Autophagy and the Ubiquitin-Proteasome System by the FoxO Transcriptional Network During Muscle Atrophy. Nat. Commun..

[53] García P.S., Cabbabe A., Kambadur R., Nicholas G., Csete M. (2010). Brief reports: Elevated myostatin levels in patients with liver disease: A potential contributor to skeletal muscle wasting. Anesth. Analg..

[54] Qiu J., Thapaliya S., Runkana A., Yang Y., Tsien C., Mohan M.L., Narayanan A., Eghtesad B., Mozdziak P.E., McDonald C. (2013). Hyperammonemia in cirrhosis induces transcriptional regulation of myostatin by an NF-κB-mediated mechanism. Proc. Natl. Acad. Sci. USA.

[55] Dasarathy S. (2017). Myostatin and Beyond in Cirrhosis: All Roads Lead to Sarcopenia. J. Cachexia Sarcopenia Muscle.

[56] Zietz B., Lock G., Plach B., Drobnik W., Grossmann J., Schölmerich J., Straub R.H. (2003). Dysfunction of the hypothalamic-pituitary- glandular axes and relation to Child-Pugh classification in male patients with alcoholic and virus-related cirrhosis. Eur. J. Gastroenterol. Hepatol..

[57] Sinclair M., Grossmann M., Hoermann R., Angus P.W., Gow P.J. (2016). Testosterone Therapy Increases Muscle Mass in Men With Cirrhosis and Low Testosterone: A Randomised Controlled Trial. J. Hepatol..

[58] Nishikawa H., Enomoto H., Ishii A., Iwata Y., Miyamoto Y., Ishii N., Yuri Y., Hasegawa K., Nakano C., Nishimura T. (2017). Elevated serum myostatin level is associated with worse survival in patients with liver cirrhosis. J. Cachexia Sarcopenia Muscle.

[59] Hanai T., Shiraki M., Watanabe S., Kochi T., Imai K., Suetsugu A., Takai K., Moriwaki H., Shimizu M. (2017). Sarcopenia Predicts Minimal Hepatic Encephalopathy in Patients With Liver Cirrhosis. Hepatol Res..

[60] Chang K.V., Chen J.D., Wu W.T., Huang K.C., Lin H.Y., Han D.S. (2019). Is Sarcopenia Associated With Hepatic Encephalopathy in Liver Cirrhosis? A Systematic Review and Meta-Analysis. J. Formos. Med. Assoc..

[61] Tsai C.F., Chen M.H., Wang Y.P., Chu C.J., Huang Y.H., Lin H.C., Hou M.C., Lee F.Y., Su T.P., Lu C.L. (2017). Proton Pump Inhibitors Increase Risk for Hepatic Encephalopathy in Patients With Cirrhosis in A Population Study. Gastroenterology.

[62] Bajaj J.S., Acharya C., Fagan A., White M.B., Gavis E., Heuman D.M., Hylemon P.B., Fuchs M., Puri P., Schubert M.L. (2018). Proton Pump Inhibitor Initiation and Withdrawal Affects Gut Microbiota and Readmission Risk in Cirrhosis. Am. J. Gastroenterol..

[63] Evans P.L., McMillin S.L., Weyrauch L.A., Witczak C.A. (2019). Regulation of Skeletal Muscle Glucose Transport and Glucose Metabolism by Exercise Training. Nutrients.

[64] Consitt L.A., Dudley C., Saxena G. (2019). Impact of Endurance and Resistance Training on Skeletal Muscle Glucose Metabolism in Older Adult. Nutrients.

[65] Pedersen B.K., Febbraio M.A. (2012). Muscles, Exercise and Obesity: Skeletal Muscle as a Secretory Organ. Nat. Rev. Endocrinol..

[66] Dagdeviren S., Jung D.Y., Friedline R.H., Noh H.L., Kim J.H., Patel P.R., Tsitsilianos N., Inashima K., Tran D.A., Hu X. (2017). IL-10 Prevents Aging-Associated Inflammation and Insulin Resistance in Skeletal Muscle. FASEB J..

[67] Lahiri S., Kim H., Garcia-Perez I., Reza M.M., Martin K.A., Kundu P., Cox L.M., Selkrig J., Posma J.M., Zhang H. (2019). The Gut Microbiota Influences Skeletal Muscle Mass and Function in Mice. Sci. Transl. Med..

[68] Tremaroli V., Bäckhed F. (2012). Functional interactions between the gut microbiota and host metabolism. Nature.

[69] Rowland I., Gibson G., Heinken A., Scott K., Swann J., Thiele I., Tuohy K. (2018). Gut microbiota functions: Metabolism of nutrients and other food components. Eur. J. Nutr..

[70] Liu T., Guo Z., Song X., Liu L., Dong W., Wang S., Xu M., Yang C., Wang B., Cao H. (2020). High-fat diet-induced dysbiosis mediates MCP-1/CCR2 axis-dependent M2 macrophage polarization and promotes intestinal adenoma-adenocarcinoma sequence. J. Cell. Mol. Med..

[71] Shimizu Y. (2018). Gut microbiota in common elderly diseases affecting activities of daily living. World J. Gastroenterol..

[72] Meijer K., de Vos P., Priebe M.G. (2010). Butyrate and other short-chain fatty acids as modulators of immunity: What relevance for health?. Curr. Opin. Clin. Nur..

[73] Walsh M.E., Bhattacharya A., Sataranatarajan K., Qaisar R., Sloane L., Rahman M.M., Kinter M., Van Remmen H. (2015). The histone deacetylase inhibitor butyrate improves metabolism and reduces muscle atrophy during aging. Aging Cell.

[74] Duan Y.H., Zeng L.M., Li F.N., Kong X.F., Xu K., Guo Q.P., Wang W.L., Zhang L.Y. (2018). β-hydroxy-β-methyl Butyrate Promotes Leucine Metabolism and Improves Muscle Fibre Composition in Growing Pigs. J. Anim. Physiol. Anim. Nutr..

[75] Hong J., Jia Y., Pan S., Jia L., Li H., Han Z., Cai D., Zhao R. (2016). Butyrate Alleviates High Fat Diet-Induced Obesity Through Activation of Adiponectin-Mediated Pathway and Stimulation of Mitochondrial Function in the Skeletal Muscle of Mice. Oncotarget.

[76] Besten G., Gerding A., van Dijk T.H., Ciapaite J., Bleeker A., van Eunen K., Havinga R., Groen A.K., Reijngoud D.J., Bakker B.M. (2015). Protection against the Metabolic Syndrome by Guar Gum-Derived Short-Chain Fatty Acids Depends on Peroxisome Proliferator-Activated Receptor γ and Glucagon-Like Peptide-1. PLoS ONE.

[77] Usami M., Miyoshi M., Kanbara Y., Aoyama M., Sakaki H., Shuno K., Hirata K., Takahashi M., Ueno K., Hamada Y. (2013). Analysis of Fecal Microbiota, Organic Acids and Plasma Lipids in Hepatic Cancer Patients With or Without Liver Cirrhosis. Clin. Nutr..

[78] Juanola O., Ferrusquía-Acosta J., García-Villalba R., Zapater P., Magaz M., Marín A., Olivas P., Baiges A., Bellot P., Turon F. (2019). Circulating Levels of Butyrate Are Inversely Related to Portal Hypertension, Endotoxemia, and Systemic Inflammation in Patients With Cirrhosis. FASEB J..

[79] Vince A., Killingley M., Wrong O.M. (1978). Effect of Lactulose on Ammonia Production in a Fecal Incubation System. Gastroenterology.

[80] Hernández M.A.G., Canfora E.E., Jocken J.W.E., Blaak E.E. (2019). The Short-Chain Fatty Acid Acetate in Body Weight Control and Insulin Sensitivity. Nutrients.

[81] Fasano A. (2011). Zonulin and Its Regulation of Intestinal Barrier Function: The Biological Door to Inflammation, Autoimmunity, and Cancer. Physiol. Rev..

[82] Fasano A. (2012). Zonulin, Regulation of Tight Junctions, and Autoimmune Diseases. Ann. N. Y. Acad. Sci..

[83] Fasano A. (2012). Intestinal Permeability and Its Regulation by Zonulin: Diagnostic and Therapeutic Implications. Clin. Gastroenterol. Hepatol..

[84] Tsukita S., Tanaka H., Tamura A. (2019). The Claudins: From Tight Junctions to Biological Systems. Trends Biochem. Sci..

[85] Biolato M., Manca F., Marrone G., Cefalo C., Racco S., Miggiano G.A., Valenza V., Gasbarrini A., Miele L., Grieco A. (2019). Intestinal Permeability After Mediterranean Diet and Low-Fat Diet in Non-Alcoholic Fatty Liver Disease. World J. Gastroenterol..

[86] Mouries J., Brescia P., Silvestri A., Spadoni I., Sorribas M., Wiest R., Mileti E., Galbiati M., Invernizzi P., Adorini L. (2019). Microbiota-driven Gut Vascular Barrier Disruption Is a Prerequisite for Non-Alcoholic Steatohepatitis Development. J. Hepatol..

[87] Fukui H. (2016). Increased intestinal permeability and decreased barrier function: Does it really influence the risk inflammation?. Inflamm. Intest. Dis..

[88] Llovet J.M., Bartolí R., Planas R., Cabré E., Jimenez M., Urban A., Ojanguren I., Arnal J., Gassull M.A. (1994). Bacterial translocation in cirrhotic rats. Its role in the development of spontaneous bacterial peritonitis. Gut.

[89] Llovet J.M., Bartolí R., March F., Planas R., Viñado B., Cabré E., Arnal J., Coll P., Ausina V., Gassull M.A. (1998). Translocated intestinal bacteria cause spontaneous bacterial peritonitis in cirrhotic rats: Molecular epidemiologic evidence. J. Hepatol..

[90] Hanai T., Shiraki M., Ohnishi S., Miyazaki T., Ideta T., Kochi T., Imai K., Suetsugu A., Takai K., Moriwaki H. (2016). Rapid Skeletal Muscle Wasting Predicts Worse Survival in Patients With Liver Cirrhosis. Hepatol. Res..

[91] Inoue T., Nakayama J., Moriya K., Kawaratani H., Momoda R., Ito K., Iio E., Nojiri S., Fujiwara K., Yoneda M. (2018). Gut Dysbiosis Associated With Hepatitis C Virus Infection. Clin. Infect. Dis..

[92] Bajaj J.S., Heuman D.M., Hylemon P.B., Sanyal A.J., White M.B., Monteith P., Noble N.A., Unser A.B., Daita K., Fisher A.R. (2014). Altered Profile of Human Gut Microbiome Is Associated With Cirrhosis and Its Complications. J. Hepatol..

[93] Bloemen J.G., Olde Damink S.W., Venema K., Buurman W.A., Jalan R., Dejong C.H. (2010). Short Chain Fatty Acids Exchange: Is the Cirrhotic, Dysfunctional Liver Still Able to Clear Them?. Clin. Nutr..

[94] Rainer F., Horvath A., Sandahl T.D., Leber B., Schmerboeck B., Blesl A., Groselj-Strele A., Stauber R.E., Fickert P., Stiegler P. (2018). Soluble CD163 and Soluble Mannose Receptor Predict Survival and Decompensation in Patients With Liver Cirrhosis, and Correlate With Gut Permeability and Bacterial Translocation. Aliment. Pharmacol. Ther..

[95] Elswefy S.E., Abdallah F.R., Atteia H.H., Wahba A.S., Hasan R.A. (2016). Inflammation, Oxidative Stress and Apoptosis Cascade Implications in Bisphenol A-induced Liver Fibrosis in Male Rats. Int. J. Exp. Pathol..

[96] Casati M., Ferri E., Azzolino D., Cesari M., Arosio B. (2019). Gut Microbiota and Physical Frailty Through the Mediation of Sarcopenia. Exp. Gerontol..

[97] Kaji K., Takaya H., Saikawa S., Furukawa M., Sato S., Kawaratani H., Kitade M., Moriya K., Namisaki T., Akahane T. (2017). Rifaximin Ameliorates Hepatic Encephalopathy and Endotoxemia Without Affecting the Gut Microbiome Diversity. World J. Gastroenterol..

[98] Bajaj J.S., Vargas H.E., Reddy K.R., Lai J.C., O’Leary J.G., Tandon P., Wong F., Mitrani R., White M.B., Kelly M. (2019). Association Between Intestinal Microbiota Collected at Hospital Admission and Outcomes of Patients With Cirrhosis. Clin. Gastrotenterol. Hepatol..

[99] Bajaj J.S., Liu E.J., Kheradman R., Fagan A., Heuman D.M., White M., Gavis E.A., Hylemon P., Sikaroodi M., Gillevet P.M. (2018). Fungal dysbiosis in cirrhosis. Gut.

[100] Yao J., Chang L., Yuan L., Duan Z. (2016). Nutrition Status and Small Intestinal Bacterial Overgrowth in Patients With Virus-Related Cirrhosis. Asia. Pac. J. Clin. Nutr..

[101] Pande C., Kumar A., Sarin S.K. (2009). Small-intestinal Bacterial Overgrowth in Cirrhosis Is Related to the Severity of Liver Disease. Aliment. Pharmacol. Ther..

[102] Zhang Y., Feng Y., Cao B., Tian Q. (2016). The Effect of Small Intestinal Bacterial Overgrowth on Minimal Hepatic Encephalopathy in Patients With Cirrhosis. Arch. Med. Sci..

[103] Bauer T.M., Steinbrückner B., Brinkmann F.E., Ditzen A.K., Schwacha H., Aponte J.J., Pelz K., Kist M., Blum H.E. (2001). Small Intestinal Bacterial Overgrowth in Patients With Cirrhosis: Prevalence and Relation With Spontaneous Bacterial Peritonitis. Am. J. Gastroenterol..

[104] Kakiyama G., Pandak W.M., Gillevet P.M., Hylemon P.B., Heuman D.M., Daita K., Takei H., Muto A., Nittono H., Ridlon J.M. (2013). Modulation of the Fecal Bile Acid Profile by Gut Microbiota in Cirrhosis. J. Hepatol..

[105] Kuno T., Hirayama-Kurogi M., Ito S., Ohtsuki S. (2018). Reduction in Hepatic Secondary Bile Acids Caused by Short-Term Antibiotic-Induced Dysbiosis Decreases Mouse Serum Glucose and Triglyceride Levels. Sci. Rep..

[106] Ramírez-Pérez O., Cruz-Ramón V., Chinchilla-López P., Méndez-Sánchez N. (2017). The Role of the Gut Microbiota in Bile Acid Metabolism. Ann. Hepatol..

[107] Sasaki T., Kuboyama A., Mita M., Murata S., Shimizu M., Inoue J., Mori K., Sato R. (2018). The Exercise-Inducible Bile Acid Receptor Tgr5 Improves Skeletal Muscle Function in Mice. J. Biol. Chem..

[108] Ridlon J.M., Kang D.J., Hylemon P.B., Bajaj J.S. (2015). Gut Microbiota, Cirrhosis, and Alcohol Regulate Bile Acid Metabolism in the Gut. Dig. Dis..

[109] Zeng Y., Chen S., Fu Y., Wu W., Chen T., Chen J., Yang B., Ou Q. (2020). Gut Microbiota Dysbiosis in Patients With Hepatitis B Virus-Induced Chronic Liver Disease Covering Chronic Hepatitis, Liver Cirrhosis and Hepatocellular Carcinoma. J. Viral. Hepat..

[110] Simbrunner B., Mandorfer M., Trauner M., Reiberger T. (2019). Gut-liver axis signaling in portal hypertension. World J. Gastroenterol..

[111] Yin M., Bradford B.U., Wheeler M.D., Uesugi T., Froh M., Goyert S.M., Thurman R.G. (2001). Reduced early alcohol-induced liver injury in CD14-deficient mice. J. Immunol..

[112] Uesugi T., Froh M., Arteel G.E., Bradford B.U., Thurman R.G. (2001). Toll-like receptor4 is involved in the mechanism of early alcohol induced liver injury in mice. Hepatology.

[113] Imajo K., Fujita K., Yoneda M., Nozaki Y., Ogawa Y., Shinohara Y., Kato S., Mawatari H., Shibata W., Kitani H. (2012). Hyperresponsivity to Low-Dose Endotoxin During Progression to Nonalcoholic Steatohepatitis Is Regulated by Leptin-Mediated Signaling. Cell. Metab..

[114] Odamaki T., Kato K., Sugahara H., Hashikura N., Takahashi S., Xiao J.Z., Abe F., Osawa R. (2016). Age-related Changes in Gut Microbiota Composition From Newborn to Centenarian: A Cross-Sectional Study. BMC Microbiol..

[115] Flemer B., Lynch D.B., Brown J.M., Jeffery I.B., Ryan F.J., Claesson M.J., O’Riordain M., Shanahan F., O’Toole P.W. (2017). Tumour-associated and Non-Tumour-Associated Microbiota in Colorectal Cancer. Gut.

[116] Zuo T., Zhang F., Lui G.C.Y., Yeoh Y.K., Li A.Y.L., Zhan H., Wan Y., Chung A., Cheung C.P., Chen N. (2020). Alterations in Gut Microbiota of Patients With COVID-19 During Time of Hospitalization. Gastroenterology.

[117] Singh S., Khan A. Clinical Characteristics and Outcomes of COVID-19 Among Patients With Pre-Existing Liver Disease in United States: A Multi-Center Research Network Study. Gastroenterology.

[118] Ren Z., Li A., Jiang J., Zhou L., Yu Z., Lu H., Xie H., Chen X., Shao L., Zhang R. (2019). Gut Microbiome Analysis as a Tool Towards Targeted Non-Invasive Biomarkers for Early Hepatocellular Carcinoma. Gut.

[119] Kim S., Covington A., Pamer E.G. (2017). The Intestinal Microbiota: Antibiotics, Colonization Resistance, and Enteric Pathogens. Immunol. Rev..

[120] Descombe J.J., Dubourg D., Picard M., Palazzin E. (1994). Pharmacokinetic study of rifaximin after oral administration in healthy volunteers. Int. J. Clin. Pharmacol. Res..

[121] Kang S.H., Lee Y.B., Lee J.H., Nam J.Y., Chang Y., Cho H., Yoo J.J., Cho Y.Y., Cho E.J., Yu S.J. (2017). Rifaximin Treatment Is Associated With Reduced Risk of Cirrhotic Complications and Prolonged Overall Survival in Patients Experiencing Hepatic Encephalopathy. Aliment. Pharmacol. Ther..

[122] Mullen K.D., Sanyal A.J., Bass N.M., Poordad F.F., Sheikh M.Y., Frederick R.T., Bortey E., Forbes W.P. (2014). Rifaximin is safe and well tolerated for long-term maintenance of remission from overt hepatic encephalopathy. Clin. Gastroenterol. Hepatol..

[123] Gangarapu V., Ince A.T., Baysal B., Kayar Y., Kılıç U., Gök Ö., Uysal Ö., Şenturk H. (2015). Efficacy of Rifaximin on Circulating Endotoxins and Cytokines in Patients With Nonalcoholic Fatty Liver Disease. Eur. J. Gastroenterol. Hepatol..

[124] Gatta L., Scarpignato C. (2017). Systematic Review With Meta-Analysis: Rifaximin Is Effective and Safe for the Treatment of Small Intestine Bacterial Overgrowth. Aliment. Pharmacol. Ther..

[125] Dalal R., McGee R.G., Riordan S.M., Webster A.C. (2017). Probiotics for people with hepatic encephalopathy. Cochrane Database. Syst. Rev..

[126] Goel A., Rahim U., Nguyen L.H., Stave C., Nguyen M.H. (2017). Systematic Review With Meta-Analysis: Rifaximin for the Prophylaxis of Spontaneous Bacterial Peritonitis. Aliment. Pharmacol. Ther..

[127] Flamm S.L., Mullen K.D., Heimanson Z., Sanyal A.J. (2018). Rifaximin Has the Potential to Prevent Complications of Cirrhosis. Therap. Adv. Gastroenterol..

[128] Kumar A., Davuluri G., Silva R.N.E., Engelen M.P.K.J., Ten Have G.A.M., Prayson R., Deutz N.E.P., Dasarathy S. (2017). Ammonia Lowering Reverses Sarcopenia of Cirrhosis by Restoring Skeletal Muscle Proteostasis. Hepatology.

[129] Schulz C., Schütte K., Vilchez-Vargas R., Vasapolli R., Malfertheiner P. (2019). Long-Term Effect of Rifaximin With and Without Lactulose on the Active Bacterial Assemblages in the Proximal Small Bowel and Faeces in Patients With Minimal Hepatic Encephalopathy. Dig. Dis..

[130] Kimer N., Pedersen J.S., Tavenier J., Christensen J.E., Busk T.M., Hobolth L., Krag A., Al-Soud W.A., Mortensen M.S., Sørensen S.J. (2018). Rifaximin Has Minor Effects on Bacterial Composition, Inflammation, and Bacterial Translocation in Cirrhosis: A Randomized Trial. J. Gastroenterol. Hepatol..

[131] Butt N.I., Butt U.I., Kakar A.A.T.K., Malik T., Siddiqui A.M. (2018). Is Lactulose Plus Rifaximin Better Than Lactulose Alone in the Management of Hepatic Encephalopathy?. J. Coll. Physicians Surg. Pak..

[132] Mekky M.A., Riad A.R., Gaber M.A., Abdel-Malek M.O., Swifee Y.M. (2018). Rifaximin Versus Metronidazole in Management of Acute Episode of Hepatic Encephalopathy: An Open Labeled Randomized Clinical Trial. Arab. J. Gastroenterol..

[133] Higuera-de-la-Tijera F., Servín-Caamaño A.I., Salas-Gordillo F., Pérez-Hernández J.L., Abdo-Francis J.M., Camacho-Aguilera J., Alla S.N., Jiménez-Ponce F. (2018). Primary Prophylaxis to Prevent the Development of Hepatic Encephalopathy in Cirrhotic Patients With Acute Variceal Bleeding. Can. J. Gastroenterol. Hepatol..

[134] Kimer N., Gudmann N.S., Pedersen J.S., Møller S., Nielsen M.J., Leeming D.J., Karsdal M.A., Møller H.J., Bendtsen F., Grønbæk H. (2018). No Effect of Rifaximin on Soluble CD163, Mannose Receptor or Type III and IV Neoepitope Collagen Markers in Decompensated Cirrhosis: Results From a Randomized, Placebo Controlled Trial. PLoS ONE.

[135] Goyal O., Sidhu S.S., Kishore H. (2017). Minimal Hepatic Encephalopathy in Cirrhosis- How Long to Treat?. Ann. Hepatol..

[136] Lauridsen M.M., Mikkelsen S., Svensson T., Holm J., Klüver C., Gram J., Vilstrup H., Schaffalitzky de Muckadell O.B. (2017). The Continuous Reaction Time Test for Minimal Hepatic Encephalopathy Validated by a Randomized Controlled Multi-Modal intervention-A Pilot Study. PLoS ONE.

[137] Lim Y.L., Kim M.Y., Jang Y.O., Baik S.K., Kwon S.O. (2017). Rifaximin and Propranolol Combination Therapy Is More Effective Than Propranolol Monotherapy for the Reduction of Portal Pressure: An Open Randomized Controlled Pilot Study. Gut Liver.

[138] Ibrahim E.S., Alsebaey A., Zaghla H., Moawad Abdelmageed S., Gameel K., Abdelsameea E. (2017). Long-term Rifaximin Therapy as a Primary Prevention of Hepatorenal Syndrome. Eur. J. Gastroenterol. Hepatol..

[139] Kimer N., Pedersen J.S., Busk T.M., Gluud L.L., Hobolth L., Krag A., Møller S., Bendtsen F. (2017). Copenhagen Rifaximin (CoRif) Study Group. Rifaximin Has No Effect on Hemodynamics in Decompensated Cirrhosis: A Randomized, Double-Blind, Placebo-Controlled Trial. Hepatology.

[140] Elfert A., Abo Ali L., Soliman S., Ibrahim S., Abd-Elsalam S. (2016). Randomized-controlled Trial of Rifaximin Versus Norfloxacin for Secondary Prophylaxis of Spontaneous Bacterial Peritonitis. Eur. J. Gastroenterol. Hepatol..

[141] Sidhu S.S., Goyal O., Parker R.A., Kishore H., Sood A. (2016). Rifaximin vs. Lactulose in Treatment of Minimal Hepatic Encephalopathy. Liver Int..

[142] Assem M., Elsabaawy M., Abdelrashed M., Elemam S., Khodeer S., Hamed W., Abdelaziz A., El-Azab G. (2016). Efficacy and Safety of Alternating Norfloxacin and Rifaximin as Primary Prophylaxis for Spontaneous Bacterial Peritonitis in Cirrhotic Ascites: A Prospective Randomized Open-Label Comparative Multicenter Study. Hepatol. Int..

[143] Zeng X., Tang X.J., Sheng X., Ni W., Xin H.G., Chen W.Z., Jiang C.F., Lin Y., Shi J., Shi B. (2015). Does Low-Dose Rifaximin Ameliorate Endotoxemia in Patients With Liver Cirrhosis: A Prospective Study. J. Dig. Dis..

[144] Mostafa T., Badra G., Abdallah M. (2015). The Efficacy and the Immunomodulatory Effect of Rifaximin in Prophylaxis of Spontaneous Bacterial Peritonitis in Cirrhotic Egyptian Patients. Turk. J. Gastroenterol..

[145] Khokhar N., Qureshi M.O., Ahmad S., Ahmad A., Khan H.H., Shafqat F., Salih M. (2015). Comparison of Once a Day Rifaximin to Twice a Day Dosage in the Prevention of Recurrence of Hepatic Encephalopathy in Patients with Chronic Liver Disease. J. Gastroenterol. Hepatol..

[146] Sharma K., Pant S., Misra S., Dwivedi M., Misra A., Narang S., Tewari R., Bhadoria A.S. (2014). Effect of Rifaximin, Probiotics, and L-Ornithine L-Aspartate on Minimal Hepatic Encephalopathy: A Randomized Controlled Trial. Saudi. J. Gastroenterol..

[147] Ali B., Zaidi Y.A., Alam A., Anjum H.S. (2014). Efficacy of Rifaximin in Prevention of Recurrence of Hepatic Encephalopathy in Patients With Cirrhosis of Liver. J. Coll. Physicians Surg. Pak..

[148] Sharma B.C., Sharma P., Lunia M.K., Srivastava S., Goyal R., Sarin S.K. (2013). A Randomized, Double-Blind, Controlled Trial Comparing Rifaximin Plus Lactulose With Lactulose Alone in Treatment of Overt Hepatic Encephalopathy. Am. J. Gastroenterol..

[149] Kalambokis G.N., Mouzaki A., Rodi M., Tsianos E.V. (2012). Rifaximin Improves Thrombocytopenia in Patients With Alcoholic Cirrhosis in Association With Reduction of Endotoxaemia. Liver Int..

[150] Sidhu S.S., Goyal O., Mishra B.P., Sood A., Chhina R.S., Soni R.K. (2011). Rifaximin Improves Psychometric Performance and Health-Related Quality of Life in Patients With Minimal Hepatic Encephalopathy (The RIME Trial). Am. J. Gastroenterol..

[151] Bajaj J.S., Heuman D.M., Wade J.B., Gibson D.P., Saeian K., Wegelin J.A., Hafeezullah M., Bell D.E., Sterling R.K., Stravitz R.T. (2011). Rifaximin Improves Driving Simulator Performance in a Randomized Trial of Patients With Minimal Hepatic Encephalopathy. Gastroenterology.

[152] Sanyal A., Younossi Z.M., Bass N.M., Mullen K.D., Poordad F., Brown R.S., Vemuru R.P., Mazen Jamal M., Huang S., Merchant K. (2011). Randomised Clinical Trial: Rifaximin Improves Health-Related Quality of Life in Cirrhotic Patients With Hepatic Encephalopathy-A Double-Blind Placebo-Controlled Study. Aliment. Pharmacol. Ther..

[153] Bass N.M., Mullen K.D., Sanyal A., Poordad F., Neff G., Leevy C.B., Sigal S., Sheikh M.Y., Beavers K., Frederick T. (2010). Rifaximin Treatment in Hepatic Encephalopathy. N. Engl. J. Med..

[154] Ohara M., Ogawa K., Suda G., Kimura M., Maehara O., Shimazaki T., Suzuki K., Nakamura A., Umemura M., Izumi T. (2018). L-Carnitine Suppresses Loss of Skeletal Muscle Mass in Patients With Liver Cirrhosis. Hepatol. Commun..

[155] Hiramatsu A., Aikata H., Uchikawa S., Ohya K., Kodama K., Nishida Y., Daijo K., Osawa M., Teraoka Y., Honda F. (2019). Levocarnitine Use Is Associated With Improvement in Sarcopenia in Patients With Liver Cirrhosis. Hepatol. Commun..

[156] Arab J.P., Martin-Mateos R.M., Shah V.H. (2018). Gut-liver Axis, Cirrhosis and Portal Hypertension: The Chicken and the Egg. Hepatol. Int..

[157] Briskey D., Heritage M., Jaskowski L.A., Peake J., Gobe G., Subramaniam V.N., Crawford D., Campbell C., Vitetta L. (2016). Probiotics Modify Tight-Junction Proteins in an Animal Model of Nonalcoholic Fatty Liver Disease. Therap. Adv. Gastroenterol..

[158] Krumbeck J.A., Rasmussen H.E., Hutkins R.W., Clarke J., Shawron K., Keshavarzian A., Walter J. (2018). Probiotic Bifidobacterium Strains and Galactooligosaccharides Improve Intestinal Barrier Function in Obese Adults but Show No Synergism When Used Together as Synbiotics. Microbiome.

[159] Sharma B.C., Singh J. (2016). Probiotics in management of hepatic encephalopathy. Metab. Brain Dis..

[160] Xia X., Chen J., Xia J., Wang B., Liu H., Yang L., Wang Y., Ling Z. (2018). Role of Probiotics in the Treatment of Minimal Hepatic Encephalopathy in Patients With HBV-induced Liver Cirrhosis. J. Int. Med. Res..

[161] Horvath A., Leber B., Schmerboeck B., Tawdrous M., Zettel G., Hartl A., Madl T., Stryeck S., Fuchs D., Lemesch S. (2016). Randomised Clinical Trial: The Effects of a Multispecies Probiotic vs. Placebo on Innate Immune Function, Bacterial Translocation and Gut Permeability in Patients With Cirrhosis. Aliment. Pharmacol. Ther..

[162] Dhiman R.K., Rana B., Agrawal S., Garg A., Chopra M., Thumburu K.K., Khattri A., Malhotra S., Duseja A., Chawla Y.K. (2014). Probiotic VSL#3 Reduces Liver Disease Severity and Hospitalization in Patients With Cirrhosis: A Randomized, Controlled Trial. Gastroenterology.

[163] Lunia M.K., Sharma B.C., Sharma P., Sachdeva S., Srivastava S. (2014). Probiotics Prevent Hepatic Encephalopathy in Patients With Cirrhosis: A Randomized Controlled Trial. Clin. Gastroenterol. Hepatol..

[164] Bajaj J.S., Heuman D.M., Hylemon P.B., Sanyal A.J., Puri P., Sterling R.K., Luketic V., Stravitz R.T., Siddiqui M.S., Fuchs M. (2014). Randomised Clinical Trial: Lactobacillus GG Modulates Gut Microbiome, Metabolome and Endotoxemia in Patients With Cirrhosis. Aliment. Pharmacol. Ther..

[165] Gupta N., Kumar A., Sharma P., Garg V., Sharma B.C., Sarin S.K. (2013). Effects of the Adjunctive Probiotic VSL#3 on Portal Haemodynamics in Patients With Cirrhosis and Large Varices: A Randomized Trial. Liver Int..

[166] Jayakumar S., Carbonneau M., Hotte N., Befus A.D., St Laurent C., Owen R., McCarthy M., Madsen K., Bailey R.J., Ma M. (2013). VSL#3^®^ Probiotic Therapy Does Not Reduce Portal Pressures in Patients With Decompensated Cirrhosis. Liver Int..

[167] Agrawal A., Sharma B.C., Sharma P., Sarin S.K. (2012). Secondary Prophylaxis of Hepatic Encephalopathy in Cirrhosis: An Open-Label, Randomized Controlled Trial of Lactulose, Probiotics, and No Therapy. Am. J. Gastroenterol..

[168] Pande C., Kumar A., Sarin S.K. (2012). Addition of Probiotics to Norfloxacin Does Not Improve Efficacy in the Prevention of Spontaneous Bacterial Peritonitis: A Double-Blind Placebo-Controlled Randomized-Controlled Trial. Eur. J. Gastroenterol. Hepatol..

[169] Pereg D., Kotliroff A., Gadoth N., Hadary R., Lishner M., Kitay-Cohen Y. (2011). Probiotics for Patients With Compensated Liver Cirrhosis: A Double-Blind Placebo-Controlled Study. Nutrition.

[170] Mittal V.V., Sharma B.C., Sharma P., Sarin S.K. (2011). A Randomized Controlled Trial Comparing Lactulose, Probiotics, and L-ornithine L-aspartate in Treatment of Minimal Hepatic Encephalopathy. Eur. J. Gastroenterol. Hepatol..

[171] Saji S., Kumar S., Thomas V. (2011). A Randomized Double-Blind Placebo Controlled Trial of Probiotics in Minimal Hepatic Encephalopathy. Trop. Gastroenterol..

[172] Malaguarnera M., Gargante M.P., Malaguarnera G., Salmeri M., Mastrojeni S., Rampello L., Pennisi G., Li Volti G., Galvano F. (2010). Bifidobacterium Combined With Fructo-Oligosaccharide Versus Lactulose in the Treatment of Patients With Hepatic Encephalopathy. Eur. J. Gastroenterol. Hepatol..

[173] Chen L.H., Huang S.Y., Huang K.C., Hsu C.C., Yang K.C., Li L.A., Chan C.H., Huang H.Y. (2019). *Lactobacillus paracasei* PS23 decelerated age-related muscle loss by ensuring mitochondrial function in SAMP8 mice. Aging (Albany NY).

[174] Carbajo-Pescador S., Porras D., García-Mediavilla M.V., Martínez-Flórez S., Juarez-Fernández M., Cuevas M.J., Mauriz J.L., González-Gallego J., Nistal E., Sánchez-Campos S. (2019). Beneficial Effects of Exercise on Gut Microbiota Functionality and Barrier Integrity, and Gut-Liver Crosstalk in an in vivo Model of Early Obesity and Non-Alcoholic Fatty Liver Disease. Dis. Model. Mech..

[175] Clarke S.F., Murphy E.F., O’Sullivan O., Lucey A.J., Humphreys M., Hogan A., Hayes P., O’Reilly M., Jeffery I.B., Wood-Martin R. (2014). Exercise and Associated Dietary Extremes Impact on Gut Microbial Diversity. Gut.

[176] Barton W., Penney N.C., Cronin O., Garcia-Perez I., Molloy M.G., Holmes E., Shanahan F., Cotter P.D., O’Sullivan O. (2018). The microbiome of professional athletes differs from that of more sedentary subjects in composition and particularly at the functional metabolic level. Gut.

[177] Huber Y., Pfirrmann D., Gebhardt I., Labenz C., Gehrke N., Straub B.K., Ruckes C., Bantel H., Belda E., Clément K. (2019). Improvement of non-invasive markers of NAFLD from an individualised, web-based exercise program. Aliment. Pharmacol. Ther..

[178] Macías-Rodríguez R.U., Ilarraza-Lomelí H., Ruiz-Margáin A., Ponce-de-León-Rosales S., Vargas-Vorácková F., García-Flores O., Torre A., Duarte-Rojo A. (2016). Changes in Hepatic Venous Pressure Gradient Induced by Physical Exercise in Cirrhosis: Results of a Pilot Randomized Open Clinical Trial. Clin. Transl. Gastroenterol..

